# Complete Blood Count Analysis on Beef Cattle Exposed to Fescue Toxicity and Rumen-Protected Niacin Supplementation

**DOI:** 10.3390/ani11040988

**Published:** 2021-04-01

**Authors:** Gaston F. Alfaro, Sandra L. Rodriguez-Zas, Bruce R. Southey, Russell B. Muntifering, Soren P. Rodning, Wilmer J. Pacheco, Sonia J. Moisá

**Affiliations:** 1 Department of Animal Sciences, Auburn University, Auburn, AL 36849, USA; gfa0002@auburn.edu (G.F.A.); muntirb@auburn.edu (R.B.M.); rodnisp@auburn.edu (S.P.R.); 2 Department of Animal Sciences, University of Illinois, Urbana, IL 61801, USA; rodrgzzs@illinois.edu (S.L.R.-Z.); southey@illinois.edu (B.R.S.); 3 Department of Poultry Sciences, Auburn University, Auburn, AL 36849, USA; wjp0010@auburn.edu

**Keywords:** fetal programming, beef cattle, fescue toxicity, rumen-protected niacin, complete blood counts, genetic test, tolerance index, animal performance, heat stress, ergovaline

## Abstract

**Simple Summary:**

Fescue toxicity results from cattle consuming fungal (*Ergot* spp.) endophyte-infected tall fescue. Ergot alkaloids like ergovaline produce vasoconstriction in cattle. Our objectives were to analyze changes in complete blood count and performance due to ergot alkaloid detoxification in growing beef cattle and the effect of selecting dams tolerant or susceptible to fescue toxicity based on their tolerance index, measured through a genetic test currently available for beef producers. Furthermore, rumen-protected niacin supplementation is proposed as a potential alleviator for vasoconstriction produced by fescue toxicity. Therefore, we assessed the effects of consuming endophyte-infected tall fescue seeds in addition to rumen-protected niacin supplementation in offspring performance and hematological parameters. Signs for anemia were noticed in susceptible heifer offspring that did not receive rumen-protected niacin, whereas inflammation or infection was detected in tolerant steers that received niacin in their diet. Typical symptoms of heat stress and intoxication with ergot alkaloids were noticed in offspring. Our results suggest that susceptible heifer offspring might have a more active detox metabolism when under fescue toxicity. Findings from this study could be utilized as a new tool to help beef cattle producers to dampen the adverse effects of fescue toxicity.

**Abstract:**

Offspring born to dams genetically tested for resistance to fescue toxicity were separated in groups based on their dams’ resistance level (tolerant vs. susceptible). Rumen-protected niacin (RPN) is proposed as a potential alleviator for vasoconstriction produced by fescue toxicity. Complete blood count (CBC) analysis was utilized for detection of significant responses to treatments applied. Our objectives were as follows: (a) to analyze changes in CBC due to fescue toxicity, maternal resistance level, and RPN in growing offspring; and (b) to assess the effects of maternal resistance level when consuming endophyte-infected tall fescue seeds in addition to RPN in offspring performance. Body weight, average daily gain, or health status were not improved by RPN or the genetic test to detect fescue toxicity resistance. Typical signs of alkaloids intoxication and heat stress were noticed in offspring. Particularly, rectal temperature was greater for susceptible control heifers. Results showed that susceptible control offspring presented signs of anemia denoted by low mean corpuscular hemoglobin (MCH) and mean corpuscular volume (MCV). High levels of white blood cells (WBC) and basophils in combination to low neutrophils to lymphocytes ratio were the signs of infection or inflammation detected in the CBC analysis, especially in tolerant niacin steers. Furthermore, offspring of control heifers had a greater percentage of reticulocytes and RDW, denoting signs of anemia.

## 1. Introduction

In the Southeastern region of the United States, cow-calf operations, the main beef production systems of the region, are forage-based production. Tall fescue (*Lolium arundinaceum (Schreb.) Darbysh*.) is a cool season perennial bunch grass with numerous positive physiological attributes, such as high nutritive value, herbage mass production, persistence, capability to be stockpiled, and pest resistance [1,2]. Most tall fescue plants establish a mutualistic symbiotic relationship with the endophyte *Epichloë coenophiala*, which is responsible for producing ergot alkaloids as second metabolites. The location of the endophyte in the plant is tightly related to the survival strategy to colonize the plant and be propagated. The most important alkaloid produced is ergovaline, which enhances plant protection against biotic and abiotic stressors [3,4,5]. Ergovaline concentration is greater in seed heads compared with vegetative organs such as leaves or steams [6]. The use of fescue seeds in research study has the advantage that animals under study can be provided with known concentrations of ergovaline in their dietary supplement, stimulating the occurrence of fescue toxicity in a controlled situation. Therefore, animals consuming endophyte-infected tall fescue experience fescue toxicity, which is characterized by a reduction in performance, elevated rectal temperature (RT) and respiration rate (RR), vasoconstriction, retention of winter hair coat, among others symptoms [7,8,9]. Nevertheless, the utilization of endophyte-free tall fescue varieties, which lack endophyte spp., leads to superior animal performance outcomes; therefore, they have been an appropriate but expensive alternative. The consumption of endophyte-infected tall fescue by gestating dams also has an effect on offspring’s performance. Shoup et al. (2016) have reported a tendency for lower adjusted 205 d weaning weight for Angus × Simmental offspring that were exposed to endophyte-infected tall fescue during the last trimester of gestation [10].

Hematological analyses, such as complete blood count (CBC), have been used extensively by researchers to assess the health status of cattle and to generate accurate disease diagnoses [11]. In a previous study, the genetic correlation and heritability of blood parameters, through CBC analysis, in crossbred beef cattle exposed to endophyte-infected and endophyte-free tall fescue was assessed [12]. However, this study accomplished its objective to report the relationship between blood-based traits and phenotypic and genotypic heritability, but it did not show differences in blood parameters for cattle exposed to endophyte-infected and endophyte-free tall fescue. In a similar study, the effect of endophyte-infected tall fescue on a limited number of blood parameters, such as glutathione, oxidized glutathione, and total glutathione on beef cows was analyzed [13]. Nevertheless, this study did not provide information about hematology disorders caused by fescue toxicity through the utilization of CBC analysis. Therefore, it is possible to state that, to the best of our knowledge, no data reporting the effects of endophyte-infected tall fescue by means of a CBC analysis on beef cattle is currently available in the literature.

The selection of animals based on their genetic resistance to fescue toxicity has been used in commercial beef production systems as a strategy for reducing economic losses caused by this syndrome. A genetic test developed to determine the level of tolerance or susceptibility to fescue toxicity (T-snip, Ag. Botanica, Columbia, MO, USA) is currently available to beef producers in the market. This commercial genetic test provides a tolerance index and the results are usually presented to producers in a six-point stars rating scale for most susceptible animals (zero stars) or most tolerant animals (five stars). The test showed promising results as a predictor of cow performance [14].

Finally, it has been broadly reported that one of the main effects of fescue toxicity on cattle is vasoconstriction [7,15,16]. Porter & Thompson (1992) have shown that in gestating ewes, vasoconstriction leads to a reduction in nutrient partitioning, causing lower performance outcomes in offspring [17]. The inclusion of dietary niacin as a vasodilator agent on livestock diets could be utilized as an alternative for damping the negative effects of fescue toxicity due to its vasoconstrictive effect in affected animals [18,19].

The objective of this study was to analyze changes in complete blood count (CBC) parameters, performance and health status in offspring’ grouped based on maternal resistance to fescue toxicity assessed with a commercially available genetic test and their supplementation with RPN for a period of 30 days after weaning.

## 2. Materials and Methods

### 2.1. Animals and Experimental Design

All the procedures for this study were conducted in accordance with a protocol approved by the Institutional Animal Care and Use Committee of Auburn University (IACUC Protocol #2019-3484). A genetic test for fescue toxicity (T-snip, Ag. Botanica, Columbia, MO, USA) was used to select animals for this study. Hair samples were collected from 256 Angus × Simmental cows by pulling approximately 20–30 hairs from the animal’s tail switch, ensuring that hair roots are present in the sample. This genetic test provides a tolerance index and results are expressed on a six-point star rating for most-susceptible animals (zero stars) or most-tolerant animals (five stars). The genetic test identified animals with low (0–1 stars, *n* = 34), medium (2–3 stars, *n* = 189), or high tolerance (4–5 stars, *n* = 33) to fescue toxicity. A total of 28 pregnant cows, out of the 256 cows genetically tested, were selected for this study based on their genetic test results (14 susceptible and 14 tolerant). At mid-gestation, selected cows were randomly assigned to a control group (CTRL), which received only base diet; or a rumen-protected niacin (RPN) group, fed with base diet and the addition of 6 gr/hd/day of top-dressed ANEVIS™ RPN (QualiTech Inc., Chaska, MN, USA) as a fixed rate for a 30-day period (from 1 July 2019 to 30 July 2019), while they received endophyte-infected tall fescue seeds. Therefore, 7 susceptible dams received a control diet (SC, *n* = 7), 7 susceptible dams received RPN top-dressed in the control diet (SN, *n* = 7), 7 tolerant dams received a control diet (TC, *n* = 7), and 7 tolerant dams received RPN in the control diet (TN, *n* = 7) during mid-gestation (~average 180 to 205 days pregnant).

After a period of approximately 10 days, dams were successfully adapted to Calan gates in order to ensure individual doses of fescue seeds and RPN. Base diet was ad libitum Bermudagrass hay in combination with a nutritional supplement composed by 1.61 kg of tall fescue seeds: 1.61 kg of pellets composed by 46.5% ground corn, 46.5% soybean meal, 5% wheat middlings, and 2% soybean oil; and 0.1 kg of molasses per animal per day (Table 1). 

Base diet was formulated to meet nutrients requirements (NRC, 2016; Table 2), and fescue seeds quantity offered was based on its ergovaline concentration. Ergot alkaloids concentration results were obtain from the Veterinary Medical Diagnostic Laboratory at the University of Missouri (Columbia, MO, USA 65211). The two lots of fescue seeds utilized in this study had an ergovaline concentration of 7300 ppb and 2700 ppb, respectively. A total of 20 µg/kg BW/day was the daily dietary dose of ergovaline offered to the animals under study to produce characteristic signs of fescue toxicity. This ergovaline concentration follows recommendations from previous studies [20,21].

Offspring calving season started on 11 September until 17 December 2019. After weaning (average 216 ± 25 days), offspring born to tolerant and susceptible dams received base diet with or without RPN and Kentucky 31 endophyte-infected tall fescue seeds (DLF Pickseed, Halsey, OR, USA) for a period of 30 days (from 16 June 2020 to 16 July 2020). Offspring steers (*n* = 19) and heifers (*n* = 9) with an average body weight (BW; 300 ± 44 kg) and age of 7–9 months-old were assigned to treatment groups based on their dam’s genetic and nutritional treatments. Therefore, this study is composed by offspring that come from susceptible dams that received a control diet (SC, *n* = 7), offspring from susceptible dams that received RPN top-dressed in the control diet (SN, *n* = 7), offspring from tolerant dams that received a control diet (TC, *n* = 7), and offspring from tolerant dams that received RPN in the control diet (TN, *n* = 7).

Offspring used for this study belongs to the beef unit located at the Auburn University Black Belt Research and Extension Center (Marion Junction, AL, USA 36759), where they have permanent access to endophyte-infected tall fescue pastures. The offspring used in this study come from 9 different sires whose genetic information in terms of fescue toxicity resistance was not evaluated. Although, sire used to breed each cow was a parameter considered at the time to select animals for this study. At weaning, offspring were relocated at the Beef Evaluation Center, Auburn University, Auburn, AL due to the accessibility to Calan gates system (Northwood, NH, USA). Calan gates were used to ensure offspring individual RPN daily dosage which was top-dressed on the based diet mixed with endophyte-infected tall fescue seeds and molasses to increase palatability.

### 2.2. Animal Performance

Individual body weight, average daily gain, respiration rate, and rectal temperature from offspring heifers and steers were obtained on Day 1 (2 weeks after weaning), 7, 14, 21, and 30 of treatment from 0600 to 0800 h from offspring heifers and steers. Average daily gain was calculated considering the difference between two body weight measurements within a known period of time. Rectal temperature data was measured using a digital rectal thermometer (Sharptemp V, Cotran Corporation, Portsmouth, RI, USA), obtaining values in Fahrenheit for posterior conversion to Celsius using the formula °C = [(°F − 32) × 5/9]. Respiration rate was recorded as the number of breaths, determined by counting flank movements, per 20 s and multiplied by 3 to obtain breaths per minute. Hair shedding score was based on visual observation of the extent of winter hair, and it was reported on a 1 to 5 scale; being 1 when the animal had removed completely the winter coat showing full shedding and a score of 5 indicating that the animal retained completely the winter coat. Hair shedding score was performed by the same trained person at Day 1 and Day 30.

### 2.3. Complete Blood Counting Analysis

Complete blood count (CBC) analysis was used as a relatively easy technique for assessing the offspring immunological status [22]. Ten mL of whole blood was obtained individually via venipuncture from the coccygeal vessels on Day 1 and Day 30. Whole blood samples were placed in BD Vacutainer^®^ Plus blood collection tubes coated with a spray-dried K2 EDTA, which works as anticoagulant (Becton Dickinson, Franklin Lakes, NJ, USA). Right after collection, samples were gently inverted several times and immediately transported to the Clinical Pathology Laboratory at the College of Veterinary Medicine of Auburn University. Complete blood count analysis was performed automatically using an ADVIA 120 Hematology System apparatus (Siemens, Munich, Germany).

### 2.4. Statistical Analyses

The response variables analyzed included body weight, body temperature, respiratory rate, average daily gain between the first and last time point, hair score, and blood parameters. The overarching linear mixed effects models used to describe all the response variables included the fixed effects of cow nutritional treatment (CTRL or RPN), genotype (tolerant or susceptible), sex, and interactions (two and third way). All response variables with the exception of ADG were analyzed assuming a repeated structure using calf as the subject. Both an unstructured and an unstructured order 1 variance–covariance structure were evaluated to accommodate for possible covariation across time points and heterogeneity of variances. The fit of both model specifications were compared using the Bayesian Information Criterion and, based on this assessment, the results from the unstructured order 1 specification are presented.

The measurement protocols required the inclusion of an additional explanatory variable to the previously described overarching model included additional explanatory variables. The models describing the profiles of body weight, body temperature, respiratory rate, and blood parameters included body weight at Day 1 as a covariate, and the time at measurement (Days 7, 14, 21, and 30) as main effect and interacting with the remaining model factors. The model describing the average daily gain excluded time because one observation was available per calf across the trial and excluded body weight as a covariate because this measurement was used to compute the response variable.

For hair score, the estimates and residual distribution from a generalized linear mixed effects model assuming a Poisson distribution and from a linear mixed effects model were consistent. In consideration of this finding, and to facilitate the interpretation of the estimates, results from the analysis of hair score using a linear mixed effects model are presented. Body weight, body temperature, respiratory rate, average daily gain, and hair score were analyzed in the observed scale. The blood parameters were transformed using a natural logarithm function to ensure that the distribution of the residuals of all variables followed a Gaussian distribution. The analysis of the mixed effect models was implemented using the MIXED procedure with the Kenward–Rogers adjustment of degrees of freedom (SAS/STAT software, Version 9.4, 2019, SAS Institute, Cary, NC, USA). The least square means, pairwise contrasts, and associated standard errors were estimated for the model factors. A significant *p* value was declared at *p* ≤ 0.05 and tendencies between *p* > 0.05 and <0.1.

## 3. Results

### 3.1. Complete Blood Count

#### 3.1.1. Neutrophil to Lymphocyte Ratio, Reticulocytes and Basophils

**Figure 1:**
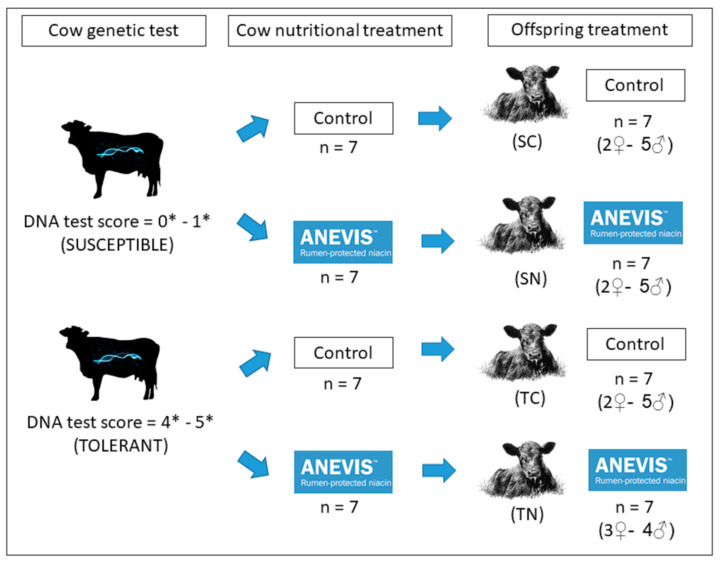
Experimental design. Abbreviations: SC: Susceptible, control; SN: Susceptible, niacin; TC: Tolerant, control; TN: Tolerant, niacin; ♀: heifers; ♂: steers, *: stars.

There was a genetic treatment × nutritional treatment × time interaction for neutrophils to lymphocytes ratio (Figure 1 and Additional Table S1). Offspring in the TN group had a greater neutrophils to lymphocytes ratio at Day 1 compared to the rest of the treatments (*p* = 0.045). In addition, there was a nutritional treatment × time interaction (*p* = 0.031) for basophils (Figure 1 and Additional Table S1). RPN offspring had a decrease in basophils concentration, while CTRL animals present an increment in basophils concentration between Day 1 and Day 30 (*p* = 0.031). Furthermore, there was a sex × time interaction (*p* = 0.002), and a nutritional treatment × time interaction for reticulocytes (*p* = 0.027). Control heifer offspring had an increment of reticulocytes percentage between Day 1 and Day 30 compared to RPN male offspring (Figure 1 and Additional Table S1).

#### 3.1.2. Hematocrit, Hemoglobin, Red Blood Cell Distribution Width, and White Blood Cells

**Figure 2:**
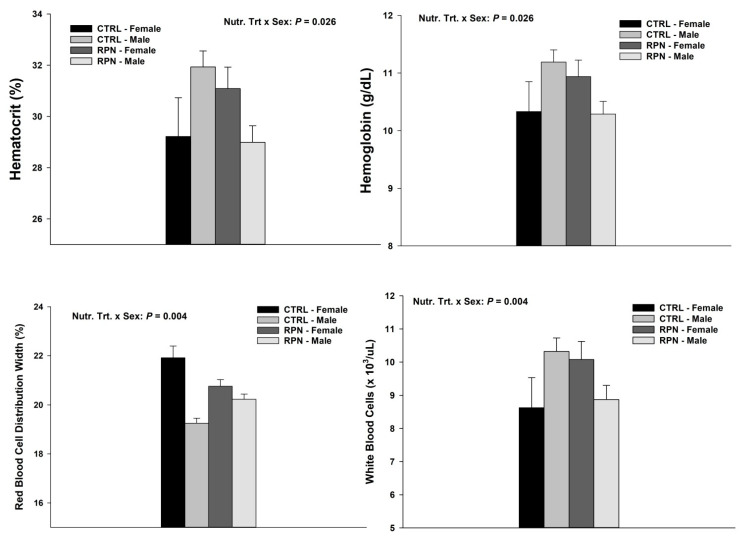
Significant nutritional treatment and sex interactions for hematocrit, hemoglobin, red blood cell distribution width, and white blood cells of Angus × Simmental steers and heifers’ offspring. Hematocrit is represented by the percentage of packed red cells in the blood, hemoglobin (g/dL), red blood cell distribution width (RDW, %), and white blood cells (×10^3^/uL) results of Angus × Simmental steers (male) and heifers (female) exposed to diets containing rumen-protected niacin (RPN), or without rumen-protected niacin (CTRL), and endophyte-infected tall fescue seeds. Statistically significant differences were declared at *p* < 0.05 and tendencies at *p* > 0.05 and <0.1.

**Figure 3:**
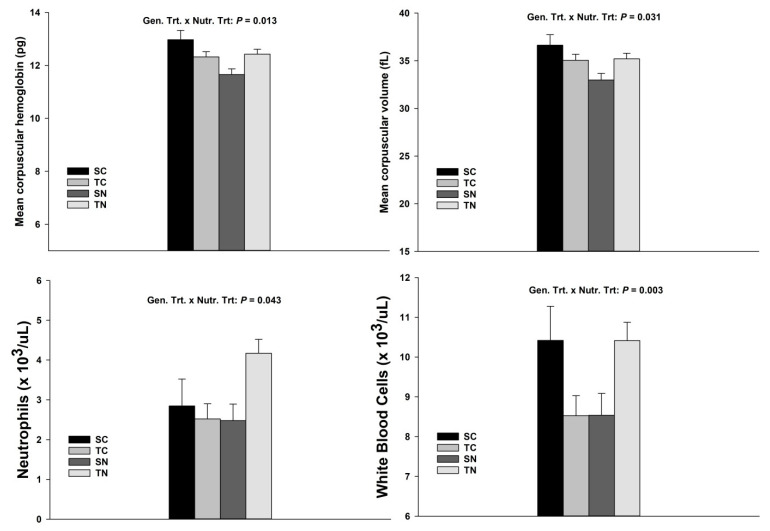
Significant genetic treatment and nutritional treatment interactions for mean corpuscular hemoglobin, mean corpuscular volume, neutrophils, and white blood cells of Angus × Simmental steers and heifers offspring. Mean corpuscular hemoglobin (MCH, pg), mean corpuscular volume (fL), neutrophils (×10^3^/uL), and white blood cells (×10^3^/uL) concentrations for Angus × Simmental offspring steers (male) and heifers (female) exposed to diets containing rumen-protected niacin (RPN) or without rumen-protected niacin (CTRL), while receiving endophyte-infected tall fescue seeds. Statistically significant differences were declared at *p* < 0.05 and tendencies at *p* > 0.05 and <0.1.

There was a nutritional treatment × sex interaction (*p* = 0.026) for hematocrit percentage and hemoglobin concentration (Figure 2 and Additional Table S1). Concentration of hemoglobin and percentage of hematocrit was greater for CTRL male offspring and RPN female offspring as compared to CTRL female and RPN male offspring. Furthermore, there was a nutritional treatment × sex interaction for red blood cell distribution width (RDW, Figure 3 and Additional Table S1). Heifer CTRL offspring had the greatest RDW dimensions (*p* = 0.004). Furthermore, there was a nutritional treatment × sex interaction (*p* = 0.004) for white blood cells (WBC) concentration (Figure 3 and Additional Table S1). CTRL male and RPN female offspring had greater WBC concentration as compared to Control female and RPN male offspring (*p* = 0.004). A tendency for greater WBC concentration was observed in susceptible heifers and tolerant steers (*p* = 0.091; Additional Table S1).

#### 3.1.3. Mean Corpuscular Hemoglobin, Mean Corpuscular Volume, Neutrophils and White Blood Cells

There was a genetic treatment × nutritional treatment interaction for mean corpuscular hemoglobin and mean corpuscular volume (*p* = 0.013 and *p* = 0.031, respectively) with greater concentrations for SC offspring and lower concentration for SN offspring (Figure 3 and Additional Table S1). There was a genetic treatment × nutritional treatment interaction (*p* = 0.043) for neutrophil with greater concentration for TN offspring. Finally, there was a genetic treatment × nutritional treatment significant interaction (*p* = 0.003) for white blood cells with greater concentrations for SC and TN offspring (Figure 3 and Additional Table S1).

#### 3.1.4. Endophyte-Infected Tall Fescue Seeds Effect on Hematocrit, Hemoglobin, Red Blood Cells, and Reticulocytes

There was a time effect (*p* < 0.01) for hematocrit, hemoglobin, and red blood cells, with lower concentrations of these parameters at the end of the experiment (Additional Figure S1 and Additional Table S1). In contrast, reticulocytes percentage increased (*p* = 0.002) during the 30-day experimental period (Additional Figure S1 and Additional Table S1). These time effects could be attributed exclusively to the administration of endophyte-infected tall fescue seeds on the experimental diet.

#### 3.1.5. Sex Effects on Red Blood Cell Distribution Width and Rumen-Protected Niacin Effect on Mean Corpuscular Hemoglobin and Mean Corpuscular Volume

Heifer offspring had greater red blood cells distribution width as compared to steers (*p* < 0.01). Furthermore, rumen-protected niacin supplementation decreased offspring mean corpuscular hemoglobin and mean corpuscular volume (*p* = 0.02 and *p* = 0.032, respectively) (Additional Figure S2 and Additional Table S1).

### 3.2. Animal Performance

#### Body Weight, Average Daily Gain, Rectal Temperature (RT), Respiration Rate (RR), and Hair Shedding Score (HS)

Additional Figure 3 shows that administration of endophyte-infected tall fescue seeds increase rectal temperature in all the animals under study, especially between Day 7 and Day 14 on treatment (*p* < 0.01). Furthermore, respiratory rate also increased for all the animals under study (*p* < 0.01), especially between Day 21 and Day 30 under study (Additional Figure S3 and Additional Table S2).

There was a genetic treatment × sex interaction for rectal temperature, with greater values for heifer offspring born to susceptible dams as compared to other treatments (*p* = 0.04). A significant nutritional treatment × sex interaction (*p* = 0.02) showed greater rectal temperatures for SC animals as compared to other treatments (Additional Figure S4 and Additional Table S2).

Body weight only had a tendency for a significant difference between heifers and steers offspring (*p* = 0.06), steers being heavier than heifers offspring (Additional Figure S4 and Additional Table S2). Furthermore, these heavier males presented a tendency for greater respiratory rates (*p* = 0.08). There were not significant differences (*p* > 0.05) for average daily gain (Additional Table S2).

Finally, hair shedding score decreased with time (*p* < 0.01) and, in general, it was lower (*p* < 0.01) for heifer offspring (Additional Figure S5 and Additional Table S1).

## 4. Discussion

### 4.1. Complete Blood Count

#### 4.1.1. Red Blood Cells Indices and Niacin

The average amount of hemoglobin per red blood cell is represented as mean corpuscular hemoglobin (MCH). In contrast, mean corpuscular volume (MCV) is a measurement of the average size of the red blood cells. A lower level of MCH and MCV was expected in animals exposed to endophyte-infected tall fescue seeds. This is mainly due to copper concentration, needed to produce hemoglobin, which is usually present in reduced concentration in animals grazing endophyte-infected tall fescue [23,24,25]. Furthermore, decrease in copper levels reduces winter coats shedding [26] or causes anemia in more severe cases [27]. During our study, in general, MCH and MCV were maintained below their lower threshold level (i.e., 14 pg for MCH and 40 fL for MCV, respectively), according to reference values for the bovine specie [28]. These results could lead us to think that offspring were slightly anemic due to fescue seeds supplementation, especially those susceptible receiving rumen-protected niacin (Figure 3 and Additional Figure S2).

Nicotinic acid is also known for its capability of forming chemical complexes with transition metals, such as iron, zinc, or manganese. The presence of nitrogen and oxygen atoms in niacin structure provides the necessary chelating site for metal ions attachment [29]. The chelating property of niacin has also a biological importance on blood parameters. Agte et al. (1997) reported an increase of blood hemoglobin levels due to the addition of dietary niacin on rats. In addition, the iron content in liver was greater for the group supplemented with niacin, which shows an improved iron bioavailability to the animal [30]. Although, our results contradict these statements.

Values of MCH typically mirror mean corpuscular volume (MCV) results, small red blood cells have a lower MCH, and vice versa. In a previous study, it was observed that niacin supplementation increase hemoglobin levels in growing turkeys [31]. Our results shows that in a fescue toxicity situation, in which animals are exposed to high concentrations of ergovaline, niacin supplementation did not exert these mentioned beneficial effects on hemoglobin concentration. Therefore, the administration of rumen-protected niacin did not have a significant effect on MCH or MCV in the animals under study.

Finally, the percentage of packed red cells in blood was within the normal range for the specie (24–46%) [28,32]. Hematocrit percentage is an indicator of oxygen carrying capacity of the circulatory system of the animal [33] and dehydration [34]. Therefore, these results indicate that our offspring did not have anemia or they were not dehydrated even though the experiment was performed during the hot summer months.

#### 4.1.2. White Blood Cells (WBC), Neutrophil:Lymphocyte Ratio, Neutrophils and Basophils

Beef steers grazing endophyte-infected tall fescue usually show reduced immunological response compared to steers grazing low-endophyte fescue [24]. A previous study showed that fescue toxicity lowers white blood cell count [35]. Our results had a similar response, showing that white blood cell number decreased in TC heifers and SN steers. The opposite response was observed for SC heifers and TN steers with greater WBC concentrations, showing signs of a negative reaction to ergovaline that leads to an activation of the innate immune system [36]. Nevertheless, further investigation will benefit the understanding of this response in animals under fescue toxicity.

**Figure 4:**
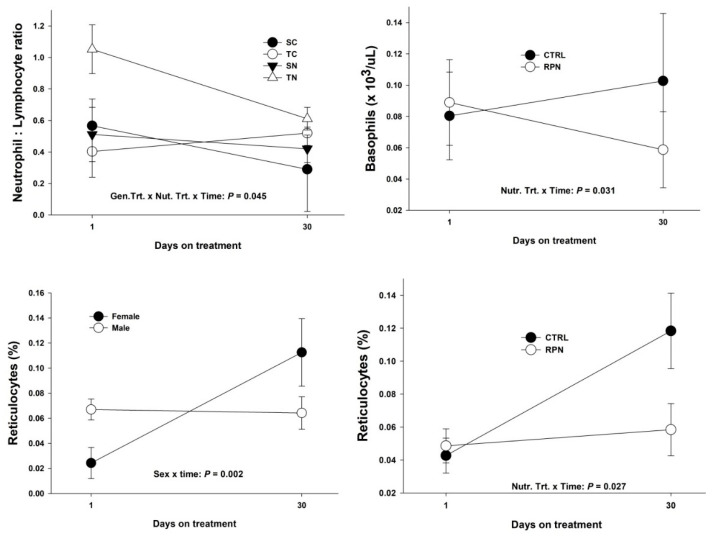
Significant interactions between genetic treatment, nutritional treatment and time for reticulocytes, basophils, and neutrophils to lymphocyte ratio for Angus × Simmental steers and heifers’ offspring. Neutrophil to lymphocyte ratio, basophils (×10^3^/uL) and percentage of reticulocytes results of Angus × Simmental steers (male) and heifers (female) exposed to diets containing rumen-protected niacin (RPN) or without rumen-protected niacin (CTRL), while receiving endophyte-infected tall fescue seeds. Statistically significant differences were declared at *p* < 0.05 and tendencies at *p* > 0.05 and <0.1.

The neutrophil:lymphocyte ratio is the number of neutrophils divided by the number of lymphocytes used as an indication of infection or inflammation [37]. Under physiological stress, the number of neutrophils increases, while the number of lymphocytes decreases [38]. The lower neutrophil:lymphocyte ratio in TN offspring might be related to the increment in neutrophil’s concentration (Figure 4). These results contradict previous research that demonstrates that nicotinic acid accelerates apoptosis in cultured neutrophils in a concentration-dependent manner [39]. Therefore, more research needs to be done in order to understand the effects of niacin in neutrophil mobilization in vivo.

Basophils are responsible for inflammatory reactions during immune response [40]. In our study, basophils concentration was above the upper threshold of 0.1 × 10^3^/uL [32] for CTRL offspring at Day 30 (Figure 4). In cattle, basophils release histamine as a consequence of a bacterial or viral infection [41]. Ergot alkaloids, including ergovaline, bind to amines (e.g., dopamine, histamine, and serotonin) and stop them from functioning, and as a result, can cause persistent vasoconstriction [42]. Therefore, we believe that the mentioned mechanism was taking place in CTRL offspring after 30 days of administration of endophyte-infected tall fescue seeds.

#### 4.1.3. Reticulocytes and Red Blood Cells Distribution Width

The toxic effect of ergovaline present in endophyte-infected tall fescue seeds could be exerted on the hematopoietic system, resulting in alterations of red blood cell function. Glucose-6-phosphate dehydrogenase deficiency is typical under these situations and, the gene controlling this enzyme is located on the X-chromosome; thus, the defect is sex-linked [43]. A high reticulocyte count and red blood cell distribution width (RDW) in CTRL heifers exposed to endophyte-infected tall fescue seeds could mean that they are producing red blood cells larger than normal. An elevated RDW is a sign of iron deficiency anemia [44], however, further research needs to be consider to explore the possibility of this condition under fescue toxicity.

### 4.2. Animal Performance

#### 4.2.1. Body Weight and Average Daily Gain

It is widely known that the consumption of endophyte-infected tall fescue leads to substantially lower individual average daily gain when compared to animals consuming non-infected pastures [2,45,46,47]. Interestingly, a meta-analysis conducted by Liebe & White (2018) reported a negative relationship between ergovaline concentration and average daily gain [5]. Furthermore, the consumption of diets based on endophyte-infected tall fescue usually leads to body weight losses in gestating animals and their offspring during growth [48,49]. In utero exposure to ergot alkaloids causes a tendency to reduce Angus × Simmental offspring’s adjusted 205 d body weight [10]. Similarly, another study indicates that lambs born to ewes receiving toxic fescue during mid and late gestation had altered growth rate [49]. Although, it was observed a decrease in individual feed intake (data not shown), body weight and average daily gain did not change due to any of the treatments applied. Nevertheless, it was recently reported that the commercially available genetic test used in this study may be considered as predictor of cow performance [14].

The offspring steers and heifers used in our study followed their previous maternal nutritional treatment, therefore; offspring presented in utero ergovaline and RPN exposure during mid to late gestation. There is no evidence reporting the effect of endophyte-infected tall fescue and RPN on growing beef cattle body weight and average daily gain. However, the addition of niacin to beef species is not well defined yet. According to a review publication, niacin supplementation levels up to 1 g/hd/day lead to an average daily gain of 72 gBW/hd/day to 82 gBW/hd/day. Any variation of average daily gain may be related to energy and protein levels in the diet [50].

#### 4.2.2. Rectal Temperature and Respiration Rate

Consumption of endophyte-infected tall fescue during summer months leads to hyperthermia as a fescue toxicity symptom. The increase in respiration rate and rectal temperature are evidence of the occurrence of hyperthermia [51]. The physiological effect of ergot alkaloids is to bind amine receptors in the peripheral blood vessels, leading to vasoconstriction and potential incapability to dissipate body heat [52,53,54]. The respiration rate increment is a physical response to reduce the rising body temperature as a consequence of fescue toxicity [55]. Several studies have reported the effect of fescue toxicity on respiration rate and rectal temperature [45,56,57]. Rectal temperature may vary depending on the animal’s sex. In a previous study, confined females have higher rectal temperature as compared to males [58]. In our study, all the animals had greater rectal temperature as compared to a reference range for calves between 38.6–39.4 °C (Additional Figure 4). Increments in body temperature due to fescue toxicity were more pronounced in SC heifers. The difference in body temperature between heifers and steers could be attributed to different hormonal levels and temperament.

To the best of our knowledge, no evidence of RPN supplementation in growing beef cattle consuming endophyte-infected tall fescue is currently available in the literature; however, RPN has been investigated in dairy cattle with conflicting performance results. Zimbelman et al. 2010 reported a significant decrease in rectal temperature in heat-stressed Holstein cows, whereas Di Constanzo et al. (1997) reported no difference for the same animal category under a similar environmental condition [59,60]. The evidence of endophyte-infected tall fescue intake during summer months supports our results, in which we observed a greater rectal temperature and respiration rate thought out the study. Therefore, these parameters may indicate that animals consuming endophyte-infected tall fescue had a limited capacity to dissipate excessive body temperature and maintain homeostasis. In addition, TN offspring had the lowest rectal temperature as compared with the other treatments, suggesting that RPN may exert its vasodilator effect producing a greater blood flow, which might helped to regulate body temperature.

#### 4.2.3. Hair Shedding Score

Rough hair coat of animals exposed to endophyte-infected tall fescue is a result of winter hair coat retention or excessive hair coat growth during long-duration days in the summer [61]. The role of ergot alkaloids in prolactin metabolism could be a possible mechanism of disruption on the follicle cycle in cattle [62]. Furthermore, ergot alkaloids are also used as a pharmacological inhibitor of pituitary hormones, such as prolactin [63]. It has been shown that prolactin plays a key role in hair growth in cattle [64] and mice [65], usually delaying or inhibiting hair regrowth. Furthermore, prolactin levels remain low on animals consuming endophyte-infected tall fescue during increasing day length; therefore, they retain winter hair coat [66]. Although, studies performed in beef cattle consuming endophyte-infected tall fescue showed rougher hair coats as compared to animals grazing pasture without fungus [26,67,68]. Our study was performed in summer months; therefore, there was a natural winter hair loss during the trial for all treatments.

Gilbert et al. (1991) investigated the effect of sire genetics, sex, diet fed, and breed on hair coat characteristics of growing Angus bulls and heifers after traditional weaning. Interestingly, even though authors found that the density (number of hairs per cm^2^) was greater for females than males; weight, length, and diameter was different in sires of different breeds [69]. We speculate that the visual estimation of hair coat at Day 1 resulted in higher values for males than females due to the greater weight, length, and diameter of hairs, not only by the appearance of presenting more hair coat, but also because they might slow down the process of shedding the winter coat.

## 5. Conclusions

In our study, the genetic test utilized on dams that provide a tolerance index to fescue toxicity, did not provide any clear result that could help to justify its implementation by beef producers to differentiate between tolerant or susceptible animals. Hematological parameters were characterized by differences between heifers, steers, and the administration of rumen-protected niacin in the supplemental diet. Results showed that susceptible control offspring presented signs of anemia denoted by low mean corpuscular hemoglobin and mean corpuscular volume after 30 days of exposure to endophyte-infected tall fescue seeds. High levels of white blood cells and basophils in combination with a low neutrophils to lymphocytes ratio were the signs of infection or inflammation detected in the complete blood count analysis, especially in tolerant niacin steers. Furthermore, offspring heifers control had a greater percentage of reticulocytes and red blood cells distribution width, denoting signs of anemia due to exposure to endophyte-infected tall fescue seeds.

Offspring from dams exposed to endophyte-infected tall fescue seeds presented the typical symptoms of high rectal temperature and respiration rate and rough hair coats. Particularly, rectal temperature was greater for susceptible control heifers. Body weight, average daily gain health related parameters were not improved by rumen-protected niacin supplementation or the genetic test to detect fescue toxicity resistance.

## Figures and Tables

**Table 1:** Composition of the diet received by dams and offspring (as fed and in dry matter basis).

Ingredients ^1^	% As-Fed	As-Fed, Kg	%DM	DM, Kg	%DM in Diet
Fescue Seeds ^2^	48.5	1.61	90.55	1.46	48.82
Pellets ^2,3^	48.5	1.61	90.59	1.46	48.84
Molasses ^2^	3	0.10	70	0.07	2.33
Total	100	3.32	251.14	2.98	100.00

^1^ All ingredients are expressed in a DM basis. ^2^ Fescue seeds, pellets, and molasses were fed as supplement on a 48.5:48.5:3 ratio. ^3^ Pellets were composed by 46.5% ground corn, 46.5% soybean meal, 5% wheat middlings, and 2% soybean oil.

**Table 2:** Nutrient composition of the diet received by dams and offspring.

Ingredients ^1^	DM	CP	NDF	ADF	TDN
Fescue Seeds ^2^	90.55	16.12	48.49	16.03	64.11
Pellets ^2,3^	90.59	29.63	12.29	4.30	74.45
Molasses ^2^	84.00	5.80	-	0.40	72.00
Bermudagrass hay	84.90	14.36	31.65	63.87	64.55

^1^ All ingredients are expressed in a DM basis. ^2^ Fescue seeds, pellets, and molasses were fed as supplement on a 48.5:48.5:3 ratio. ^3^ Pellets were composed by 46.5% ground corn, 46.5% soybean meal, 5% wheat middlings, and 2% soybean oil.

## Data Availability

The following are available online at https://https://zenodo.org/record/4564620#.YDj_kWhKhaQ (accessed date 31 January 2021).

## Supplementary Materials

The following are available online at https://www.mdpi.com/article/10.3390/ani11040988/s1, Additional Figure S1. Time effect for hematocrit, hemoglobin, red blood cells and, reticulocytes; Additional Figure S2. Sex effect for red blood cells distribution width and nutritional treatment effect for mean corpuscular hemoglobin and mean corpuscular volume. Additional Figure S3. Time effect for rectal temperature and respiration rate. Additional Figure S4. Significant interactions for rectal temperature and sex effect for body weight and respiration rate. Additional Figure S5. Time effect and sex effect for hair shedding score. Additional Table S1. Complete blood count parameters and hair shedding score results from statistical analysis. Additional Table S2. Average daily gain, body weight, rectal temperature and respiration rate results from statistical analysis.
