# Supplementary material for: Complete Blood Count Analysis on Beef Cattle Exposed to Fescue Toxicity and Rumen-Protected Niacin Supplementation
Source: Animals (Basel). 2021 Apr 1;11(4):988. doi: 10.3390/ani11040988 (PMC8065407; doi:10.3390/ani11040988)
Supplement: Supplementary file 1 [file animals-11-00988-s001.zip › animals-1113527 supplementary.docx]

Supplementary Materials

Complete Blood Count Analysis on Beef Cattle Exposed to Fescue Toxicity and Rumen-Protected Niacin Supplementation

Gaston F. ****Alfaro**** ^1^, Sandra L. Rodriguez-Zas ^2^, Bruce R. Southey ^2^, Russell B. Muntifering ^1^, Soren P. Rodning ^1^, Wilmer J. Pacheco ^3^ and Sonia J. Moisá ^1,^*


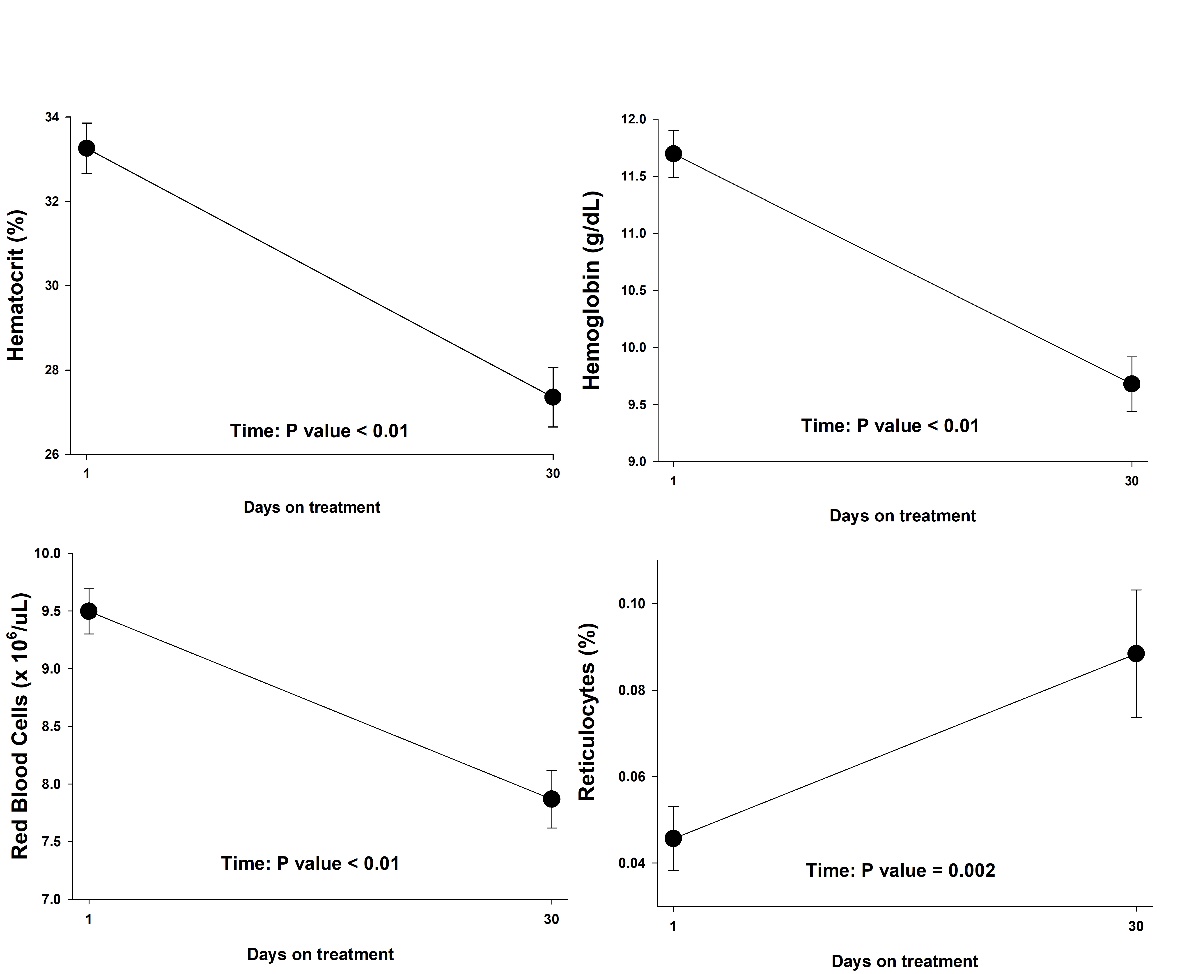


**Additional Figure S1.** Time effect for hematocrit, hemoglobin, red blood cells and, reticulocytes during the 30-day treatment period for Angus × Simmental steers (male) and heifers (female) exposed to diets containing rumen-protected niacin (RPN) or without rumen-protected niacin (CTRL) and endophyte-infected tall fescue. Statistically significant differences were declared at *P* < 0.05 and tendencies at *P* > 0.05 and < 0.1.


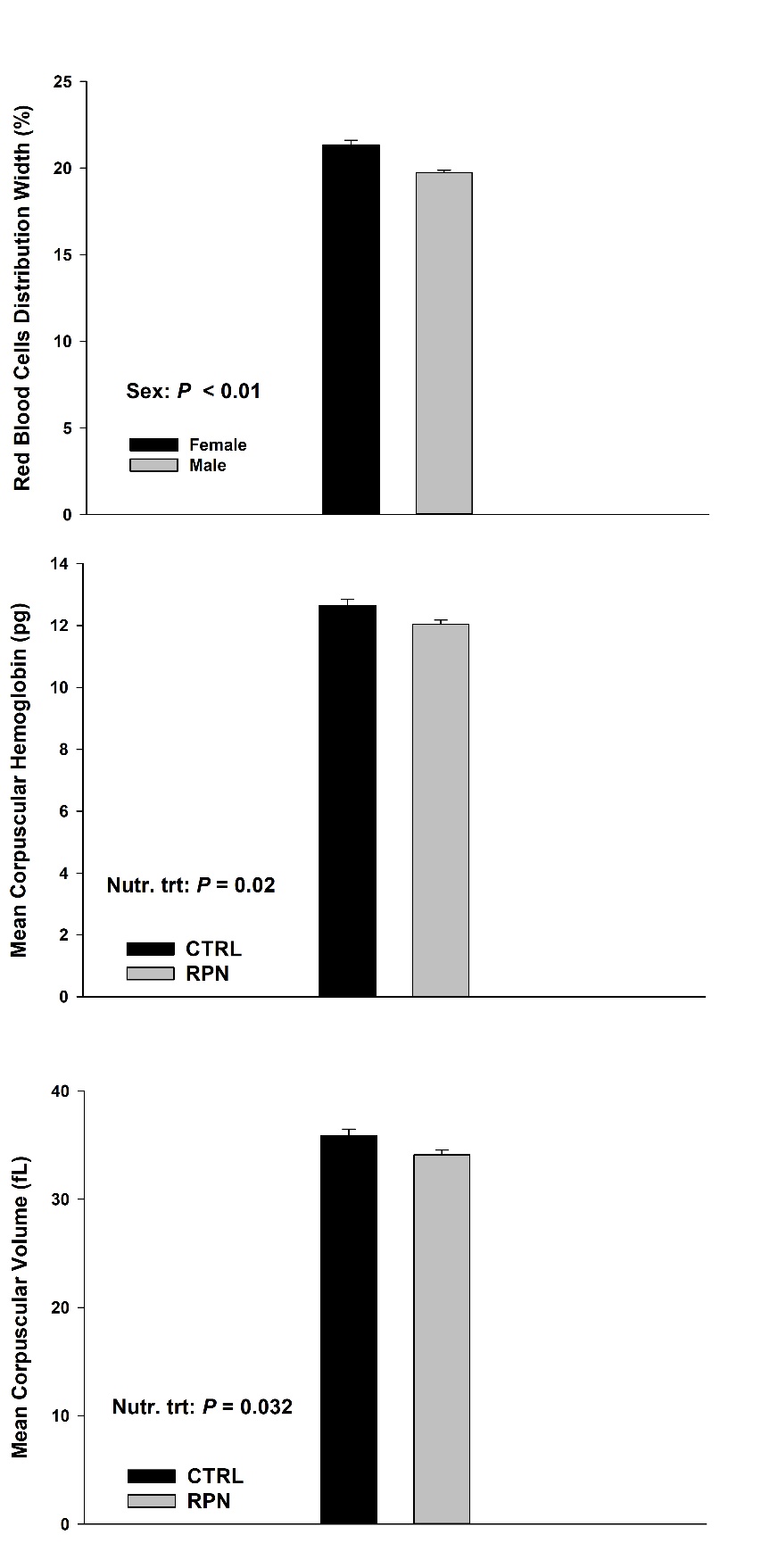


**Additional Figure S2.** Sex effect for red blood cells distribution width and nutritional treatment effect for mean corpuscular hemoglobin and mean corpuscular volume during the 30-day treatment period for Angus × Simmental steers (male) and heifers (female) exposed to diets containing rumen-protected niacin (RPN) or without rumen-protected niacin (CTRL) and endophyte-infected tall fescue. Statistically significant differences were declared at *P* < 0.05 and tendencies at *P* > 0.05 and < 0.1.


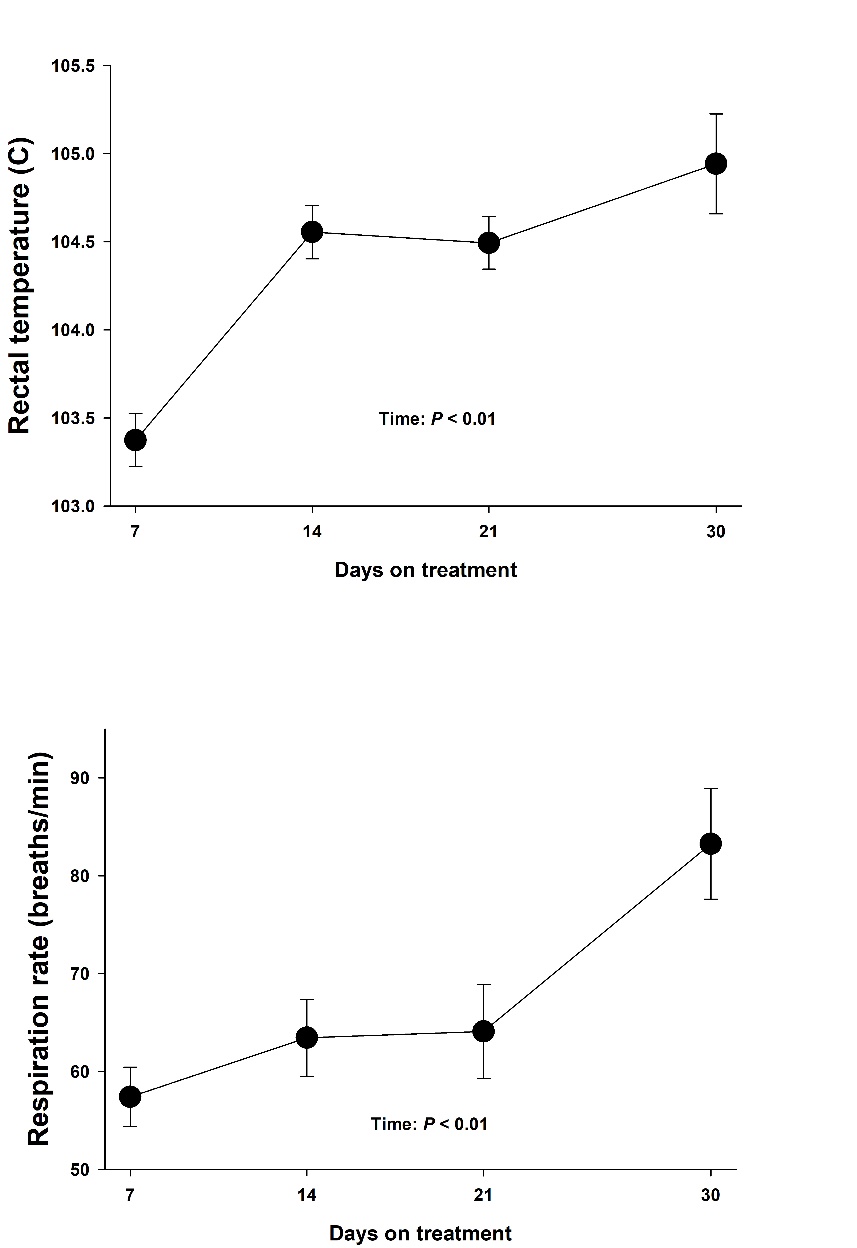


**Additional Figure S3.** Time effect for rectal temperature and respiration rate during the 30-day treatment period for Angus × Simmental steers (male) and heifers (female) exposed to diets containing rumen-protected niacin (RPN) or without rumen-protected niacin (CTRL) and endophyte-infected tall fescue. Statistically significant differences were declared at *P* < 0.05 and tendencies at *P* > 0.05 and < 0.1.


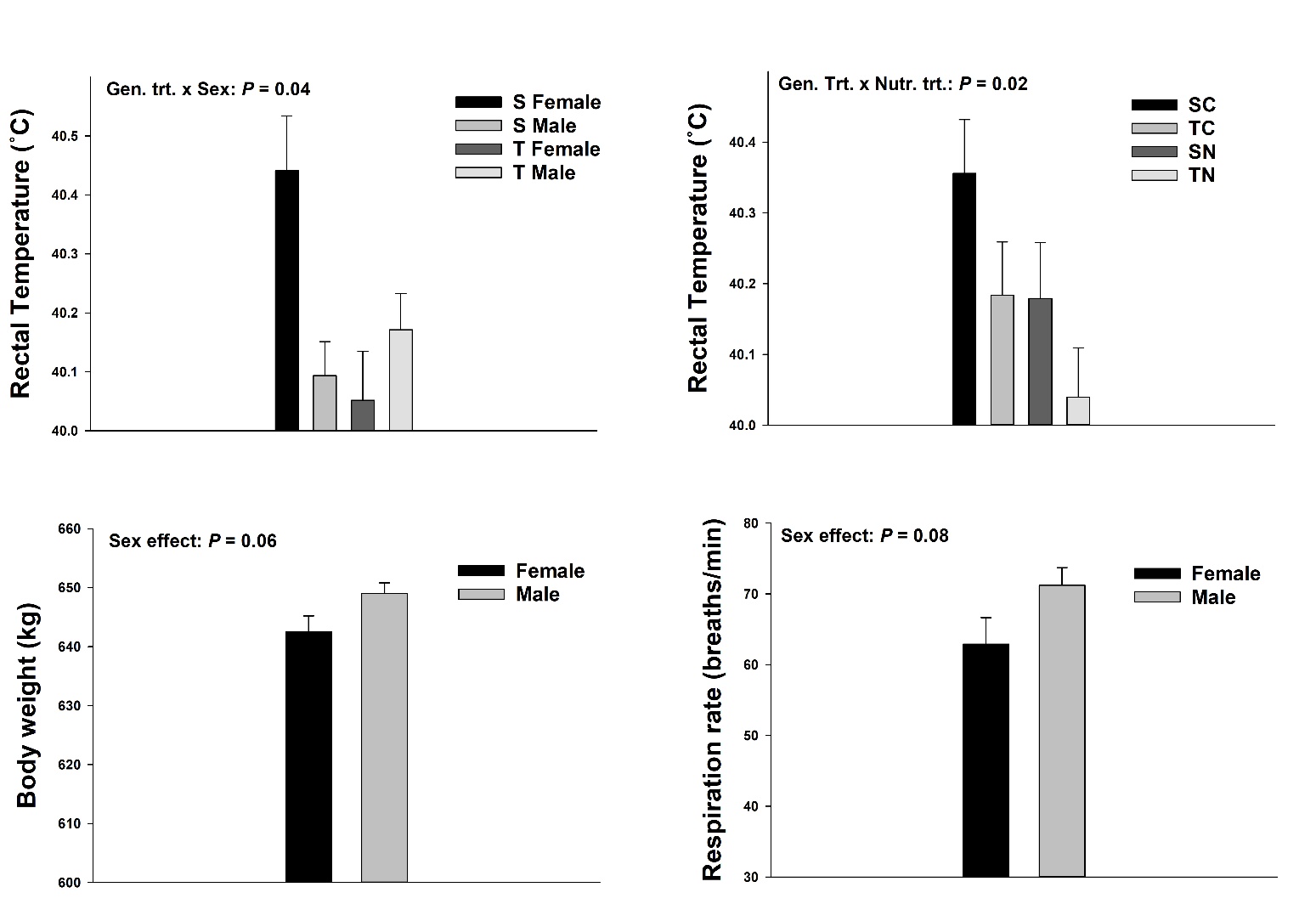


**Additional Figure S4.** Significant interactions for rectal temperature and sex effect for body weight and respiration rate during the 30-day treatment period for Angus × Simmental steers (male) and heifers (female) exposed to diets containing rumen-protected niacin (RPN) or without rumen-protected niacin (CTRL) and endophyte-infected tall fescue. Statistically significant differences were declared at *P* < 0.05 and tendencies at *P* > 0.05 and < 0.1.


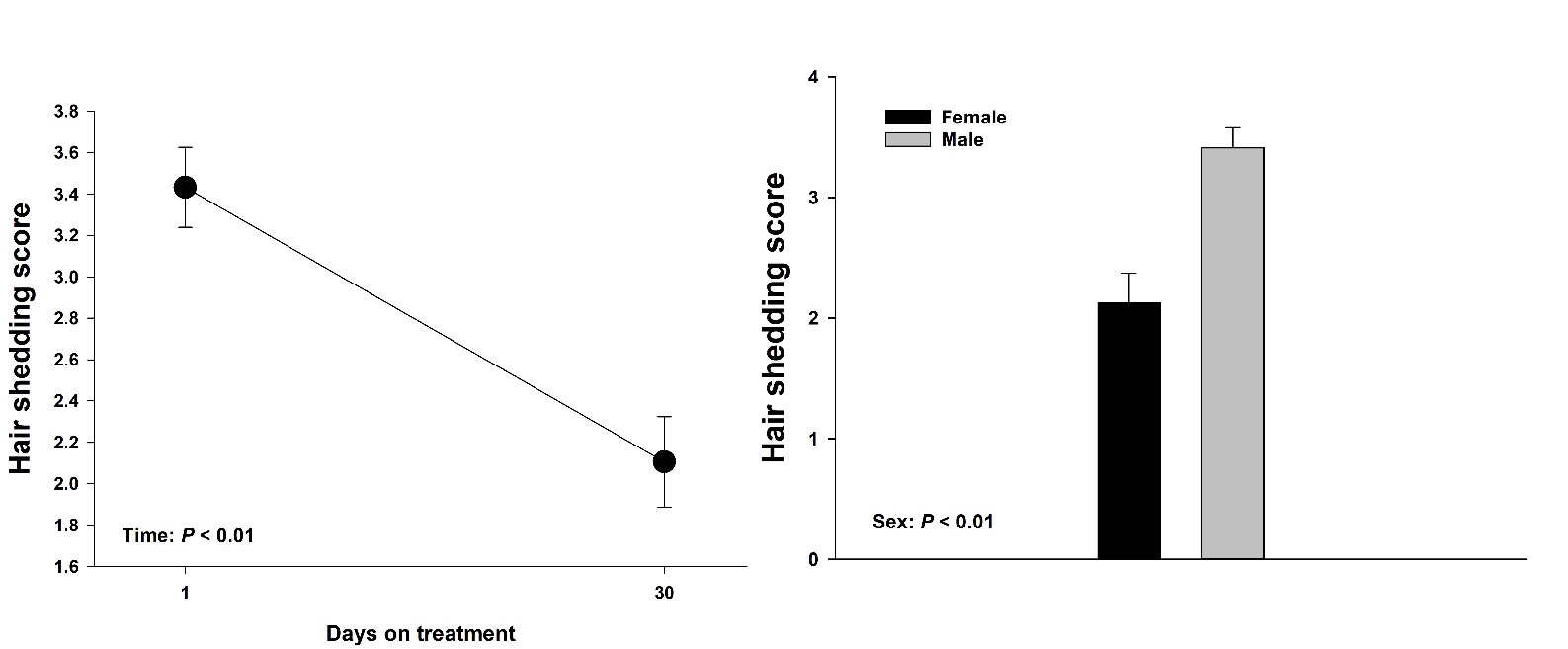


**Additional Figure S5.** Time effect and sex effect for hair shedding score for Angus × Simmental steers (male) and heifers (female) exposed to diets containing rumen-protected niacin (RPN) or without rumen-protected niacin (CTRL) and endophyte-infected tall fescue during the 30-day treatment period. Statistically significant differences were declared at *P* < 0.05 and tendencies at *P* > 0.05 and < 0.1.

**Additional Table S1.** Complete blood count parameters and hair shedding score results from statistical analysis.

**Additional Table S2.** Average daily gain, body weight, rectal temperature and respiration rate results from statistical analysis.
